# Exploring Ayurveda's Potential in Parkinson's Disease: A Comprehensive Narrative

**DOI:** 10.7759/cureus.93992

**Published:** 2025-10-06

**Authors:** Umesh Chikkanna, Shivakumar Venkatram, Bharath Holla, Rohan R Mahale, Hemant Bhargav, Lakshmi Nishitha Jasti, Kishore Kumar Ramakrishna, Shivarama Varambally

**Affiliations:** 1 Department of Integrative Medicine, National Institute of Mental Health and Neurosciences, Bengaluru, IND; 2 Department of Neurology, National Institute of Mental Health and Neurosciences, Bengaluru, IND; 3 Department of Psychiatry, National Institute of Mental Health and Neurosciences, Bengaluru, IND

**Keywords:** ayurveda, kampavāta, mucuna pruriens, panchakarma, parkinson's disease

## Abstract

Parkinson's Disease (PD) is a protracted and progressive neurodegenerative disorder, bearing a notable semblance to the Ayurvedic construct of *kampavāta* (trembling or shaking, often meaning a neurological disorder such as PD). This comprehensive review endeavours to offer insight into the Ayurvedic comprehension of PD and subsequently appraise the available clinical literature, explicitly investigating the utilization of Ayurvedic approaches in PD management. The methodology involved scrutinizing classical Ayurveda texts and meticulously examining pertinent scientific databases, including PubMed, Google Scholar, and the AYUSH Research Portal. This quest employed MeSH (Medical Subject Headings) terms and keywords to identify clinical trials employing Ayurvedic medicine/therapy as an intervention within the context of PD. The eligibility encompassed clinical trials published from January 2000 to December 2020; exclusions comprised review articles, conceptual papers, editorials, and studies published in languages other than English. A total of 126 studies were screened for selection criteria, and seven were included after removing duplicates. Among the seven included studies, six showed the beneficial effects of an herb called *Mucuna pruriens*, either as a stand-alone or in combination with other herbs, and one trial highlighted the significance of bio-cleansing along with *M. pruriens*. Duration of intervention ranged from assessment of acute effects to 56 weeks. The synthesis of the reviewed data showed that *M. pruriens* exhibited an extended ON state, effectively ameliorating motor symptoms even among individuals afflicted with advanced PD while manifesting a favourable side effect profile. Nonetheless, it is imperative for future investigations to scrutinize Ayurvedic interventions in concordance with classical texts, incorporating longer follow-up periods and objective parameters to enhance the comprehensiveness and rigor of the evidence base for Ayurvedic interventions for PD.

## Introduction and background

Background

Parkinson's disease (PD) is a chronic and progressive neurodegenerative movement disorder within the purview of neurology [1]. This disorder arises from the depletion of dopamine within the basal ganglia due to the degeneration of dopaminergic neurons, culminating in classical Parkinsonian motor manifestations such as bradykinesia, tremor, rigidity, and postural instability [2]. The affliction's prevalence is around 1% of the population aged 65 years and above, displaying a male-to-female ratio of 2:1 [3]. In 2016 alone, PD accounted for a staggering 3.2 million disability-adjusted life years (DALYs) and 211,296 fatalities (95% uncertainty interval: 167,771-265,160) [4,5]. In light of the Global Burden of Disease Study (GBD), the estimated global PD population reached 9.4 million in 2020, surpassing the previously recorded six million cases in 2016 [6].

Intriguingly, investigations into the prevalence of PD in India remain scarce. A comprehensive household survey conducted in 2004 unveiled a crude prevalence rate of 33 per 100,000 individuals, escalating to 76 per 100,000 upon age adjustment, revealing the prevalence of Parkinsonism within the Bengaluru district in South Karnataka, India [7]. Parallel endeavours discovered prevalence rates of 45.82 per 100,000 in a Kolkata-based study in 2006, while a distinct study involving a cohort of 14,010 Parsis within colonies in Mumbai, Western India, indicated a remarkably elevated crude prevalence rate of 328.3 per 100,000 [8-10].

The contemporary framework of PD management predominantly revolves around pharmacological intervention, although its efficacy wanes as the disease advances, often entailing distressing side effects like motor fluctuations and dyskinesias [11]. Although productive, the recourse of deep brain stimulation remains financially prohibitive for most patients. Interestingly, it has been postulated that nearly half of PD patients in India use complementary and alternative medicine (CAM), with prolonged disease duration and levodopa usage correlating with heightened CAM adoption, notably encompassing Ayurveda as a frequently opted modality [12]. This study aimed to delve into the Ayurvedic comprehension of PD and scrutinize studies on Ayurvedic interventions for PD, thereby elucidating strategies for evidence-based management.

PD in Ayurveda

*Vāta* (combination of primordial elements air and space), one of the principal bio-forces in Ayurveda, plays a pivotal role in regulating neurological functions and coordinating the body’s neural activities. PD can be comprehended within the paradigm of *vāta vyādhi* (neurological disorders) as delineated in Ayurveda. *Vāta vyādhi* manifests either due to *dhātukṣaya* (degeneration of tissue elements) or *mārgāvaraṇa* (hindrance to normal *vāta* functions) [13]. General clinical features that typify *vāta vyādhi*, such as *kampa* or *vepathu* (tremor), *stambha* (rigidity), and *gatisaṅga* or *ceṣṭāsaṅga* (bradykinesia), exhibit remarkable parallels with the motor symptoms characteristic of PD. Notably, *vepathu* emerges explicitly when the *shiras* (head) is affected due to *vāta* derangement or instances of *śiromarmābhighāta* (head injuries/lesions of the central nervous system arising from intrinsic or extrinsic factors) [14,15].

More specifically, the clinical manifestations of PD find close correlation with kampavāta, a subtype of *vāta vyādhi* characterized by *kara pāda-tala kampa* (tremors of the limbs), *deha bhrama duḥkhite* (disturbance of gait and posture), *nidrā-bhaṅga* (sleep disturbances), and *mati-kṣīṇa* (cognitive decline) [16]. Additionally, when *vyana vata*, the subtype of vāta responsible for coordinating motor activity, is obstructed by *kapha*, the biological force that provides strength and structural integrity, the resulting condition, termed *kaphāvṛta vyāna* (impediment of *vyāna vāta* by *kapha*), manifests clinically as bradykinesia, *svaragraha* (hoarseness of voice), rigidity, and tremors [14]. While *kampavāta *is predominantly characterized by tremors due to the exclusive involvement of *vāta*, *kaphāvṛta vyāna* is marked by akinesia and gait freezing, reflecting the combined interplay of *kapha* and *vāta*.

## Review

Methodology 

A comprehensive search was conducted within the electronic databases of PubMed, AYUSH research portal, and Google Scholar, encompassing the time frame between January 2000 and December 2020. Only clinical studies involving Ayurveda single/polyherbal/*panchakarma* (bio-cleansing) intervention published in the English language were included*. *Pilot studies, systematic reviews, preclinical studies, case studies, and unpublished manuscripts were excluded. The scope of the exploration was further broadened to include all available research works accessible through the AYUSH research portal, an initiative of the Ministry of AYUSH, Government of India [17]. The search protocol within the AYUSH research portal was stratified, incorporating successive filters including (a) Medical system: Ayurveda, followed by (b) Category: Clinical research, yielding a total of 2921 studies. Further refinement ensued through applying (c) Body system: Neurological, and (d) Disease: PD parameters. This refined dataset identified a compilation of 12 studies stratified as Grades A (meta-analysis/randomized controlled trials), B (non-randomized trials), and C (good quality clinical reports). Specifically, this encompassed three studies classified under Grade A, along with one study under Grade B, and nine studies under Grade C. Among the collected corpus, one study was an RCT, three were randomized uncontrolled trials, six were case studies, and two reports undertook a pharmacokinetic and pharmacodynamic exploration.

PubMed was searched using a combination of relevant MeSH (Medical Subject Headings) terms and keywords [((Parkinson*[tiab] OR kampavāta[tiab] OR tremor[tiab]) AND (Ayurveda*[tiab] OR mucuna[tiab] OR panchakarma[tiab] OR bio cleansing*[tiab]))] for clinical trials involving Ayurveda drug/therapy as intervention in PD.

**Figure 1:**
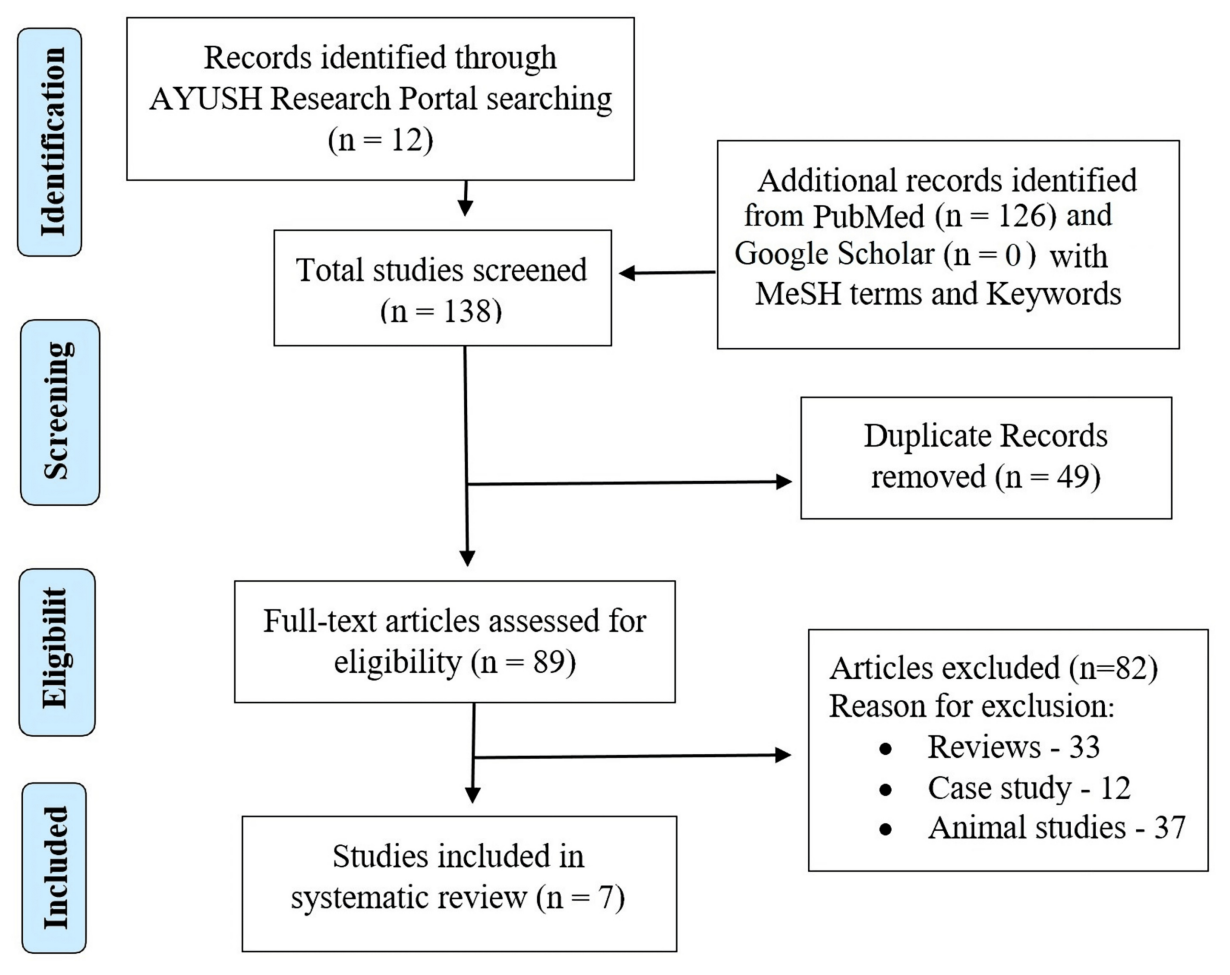
PRISMA flow diagram PRISMA: Preferred Reporting Items for Systematic Reviews and Meta-Analyses

A total of 126 studies were obtained and screened for selection criteria, and after removing duplicates, seven studies were included for review. Google Scholar was employed to supplement PubMed by capturing additional relevant studies, particularly those related to Ayurveda that may be published in non-indexed journals, theses, conference proceedings, and other forms of grey literature. However, no additional studies were found in Google Scholar. The selection procedure is shown in the Preferred Reporting Items for Systematic Reviews and Meta-Analyses (PRISMA) flow chart (Figure 1). The details of the studies included in this review are summarized in Table 1.

**Table 1:** Clinical trials involving Ayurveda intervention in Parkinson's disease “ON” state refers to the period when medication, especially levodopa, is working optimally, and the patient experiences good control of motor symptoms UPDRS: Unified Parkinson's Disease Rating Scale; LD: levodopa; CD: carbidopa; AIMS: Abnormal Involuntary Movement Scale; PDQ-39: Parkinson's Disease Questionnaire 39-item; MDS: Movement Disorder Society; NMSQ: Non-motor Symptoms Questionnaire

Author (Year)	Design	Intervention details	Control	Outcome measures	Results
Vaidya et al., 1978 [18]	Open-label clinical trial (n = 23)	Oral administration of *Mucuna pruriens* 15 G four times a day	None	Morbidity score consisting of Northwestern University Disability Scores (NUDS) Physical signs score.	*M. pruriens* dose was well tolerated, and the therapeutic response was statistically significant.
Sivaramakrishna et al., 1990 [19]	Open-label clinical trial. (n = 16)	Oral administration of vāruṇi taila, ātmaguptādi rasa, māṣadi kvātha for 45 days along with three courses of seven days māṣadi kvātha nasya karma (Errhine).	None	Symptoms of PD.	Marked reduction in rigidity, gait disturbance, and tremor.
Nagashayana 2000 [20]	Open-label Uncontrolled clinical study (n = 18)	Group A – Cleansing therapy – Duration 28 days, followed by a concoction of *Mucuna pruriens*, *Hyoscyamus reticulatis*, *Withania somnifera*, and *Sida cordifolia*. Duration – 56 days. Group B - Concoction of *M. pruriens*, *H. Reticulatis*, *W. somnifera*, and *S. cordifolia*. Duration – 84 days.	None (two active intervention groups)	UPDRS Score	Activities of daily living and UPDRS showed significant improvement in Group A compared to Group B.
Manyam et al., 2004 [21]	Open-label Uncontrolled clinical study (n = 60)	*Mucuna pruriens* 7.5 grams, thrice daily. Duration - 12 weeks	None	UPDRS Score	Hoehn and Yahr (H&Y) stage and UPDRS scores showed significant reductions.
Katzenschlager et al., 2004 [22]	Double-blind, randomized crossover trial (n = 8)	Single dose of LD/CD (200/50 mg) and *Mucuna pruriens* (15 and 30 grams) at weekly intervals for measuring acute effects.	None	UPDRS, tapping speed, modified AIMS, and Goetz scales	Faster onset of the anti‐Parkinsonian effects of *M. pruriens* with no significant differences in dyskinesias or tolerability
Cilia et al., 2017 [23]	Noninferiority, DB, crossover, phase 2b RCT (n = 18)	(1) Combination of levodopa (3.5 mg/kg) and dopa-decarboxylase inhibitor benserazide. (2) High-dose (17.5 mg/kg) of *Mucuna pruriens*. (3) Low-dose (12.5 mg/kg) of *M. pruriens*. (4) Levodopa without dopa-decarboxylase inhibitor benserazide (17.5 mg/kg). (5) *M. pruriens* with benserazide (3.5 mg/kg). (6) placebo. For measuring acute effects.	Placebo	UPDRS-III	Longer ON state and more significant improvement with high-dose *M. pruriens*.
Cilia et al., 2018 [24]	Open-label, noninferiority, randomized, crossover, phase-2b pilot trial (n=14)	*Mucuna pruriens* and LD/CD.	None (two active intervention groups)	PDQ-39 MDS-UPDRS NMSQ Time with good mobility without troublesome dyskinesias as measured by 24-hour home diaries.	*M. pruriens* powder was able to control motor and non-motor symptoms in patients with advanced PD.

Results

Out of seven studies, six showed beneficial effects of an herb, *Mucuna pruriens *(a standard ayurvedic treatment for *kampavata*), either as a stand-alone or in combination with other herbs [18,20-24], and one trial highlighted the significance of bio-cleansing along with *M. pruriens* [19]. The studies on *M. pruriens* alone were either efficacy and safety studies or studies examining the acute ON effects compared to levodopa/carbidopa or levodopa/benserazide (“ON” refers to the period when medication, especially levodopa, is working optimally, and the patient experiences good control of motor symptoms) [18,20-24]. Of these six studies, three examined the efficacy and safety of *M. pruriens* [18,21,23]. The duration of *M. pruriens* administration in these efficacy and safety assessments ranged from a minimum of four weeks to a maximum of 20 weeks. Dosing regimens varied from 7.5 grams, administered thrice daily, to 15 grams four times per day. Attaining optimal symptom control required 5±2 sachets (each containing 7.5 grams) for levodopa-naïve patients and 7±3 sachets for those who had discontinued levodopa/carbidopa. The levodopa content within each 7.5-gram sachet maintained a 33.33 mg/g concentration [21]. Notably, one study reported subjective motor performance comparable to or even superior to levodopa/carbidopa, despite tolerability concerns associated with *M. pruriens* [20].

The efficacy of *M. pruriens* was evaluated with various assessment tools, including Northwestern University Disability Scores (NUDS) [25], Unified Parkinson's Disease Rating Scale (UPDRS) [26], Parkinson's Disease Questionnaire (PDQ-39) [27], Non-motor Symptoms Questionnaire (NMSQ) [28], and 24-hour home diaries recording good mobility without troublesome dyskinesias. Adverse effects of *M. pruriens*, although infrequent and mild, were dose-dependent and encompassed symptoms such as giddiness, sweating, flatulence, diarrhoea, dry mouth, rash, pruritus, and blue-black urine [18].

Two studies probed the acute effects and dyskinesia profiles of *M. pruriens* compared to levodopa/carbidopa across varying dosages [22,23]. At a dose of 30 g,* M. pruriens* precipitated a significantly faster onset of anti-Parkinsonian effects (34.6 vs. 68.5 minutes), with no discernible disparities in tolerability or dyskinesias relative to levodopa/carbidopa. Conversely, another investigation revealed that a higher dose of *M. pruriens* (17.5 mg/kg) induced more pronounced amelioration of motor symptoms (UPDRS-III) at 90 and 180 minutes, extending the ON state duration by 25% compared to levodopa/carbidopa [24].

In addition to *M. pruriens*, the efficacy of bio-cleansing techniques (*panchakarma* procedures) was investigated in conjunction with palliative therapy across two studies [19,20]. One study solely employed the *nasya* (nasal instillation) procedure, while the other systematically integrated a repertoire of bio-cleansing interventions, including *snehana* (oleation), *svedana* (sudation), *virechana* (purgation), *basti* (enema), and *nasya*. The findings exhibited significant enhancements in daily activities and UPDRS scores among patients subjected to panchakarma followed by palliative care, in contrast to those undergoing solely palliative care [20].

Discussion

In the Indian context, a notable proportion (approximately 46%) of PD patients reported using CM; it was also seen that CAM use was more common among patients with longer disease duration, higher education (graduates and above), urban residence, and a fairly good perception of their own health [12]. Ayurveda, distinguished for its holistic approach, tailored treatment regimens, and comprehensive engagement with psychosomatic well-being, bears relevance. Given PD's doctrinal association with *vāta vyādhi *(specifically *kampavāta* or *kaphāvṛta* *vyāna*), its therapeutic management revolves around measures aimed at mitigating the deranged *vāta dosha*, including oleation, sudation, intestinal purgation, therapeutic enema, and nasal installations [29]. These practices are subsequently accompanied by administering singular or composite herbal formulations as substantiated efficacious interventions. Predominantly, the research landscape has concentrated on investigating the efficacy of *M. pruriens* as an interventional agent, either in isolation or in synergistic conjunction with other pharmacotherapies. Notably, a solitary trial has ventured into integrating *panchakarma* techniques and harmonizing therapeutic agents, aligning with the tenets of classical Ayurvedic management for PD [20]. This focus reflects the need to understand Ayurvedic interventions described in classical texts and to guide future research. Such efforts may help clarify the possible biological mechanisms underlying these treatments.

M. pruriens and Entourage Effect

*M. pruriens* is most commonly used in treating *kampavāta*, either as monotherapy or in combination with other herbs. The clinical effects have been shown in studies eliciting its acute ON and long-term clinical outcomes [22,24]. Studies suggest that *M. pruriens* acts through a novel mechanism different from levodopa. The natural form of levodopa contained in *M. pruriens* is in combination with other biological agents that work synergistically with levodopa and prevent it from rapid decarboxylation/acting as an intrinsic dopa decarboxylase inhibitor (DDCI)-like activity/natural anti-dyskinetic agents that prevent dyskinesia [30]. Phytochemicals like alkaloids, alkylamines, arachidic acid, beta-carboline, harmine, ufotenine, dopamine, flavones, galactose, gallic acid, genistein, glutathione, hydroxygenistein, 5-hydroxytryptamine, N, N-dimethyltryptamine (DMT), 5-methoxy-dimethyltryptamine (5-MeODMT), 6-methoxyharman, mucunadine, mucunain, mucunine, myristic acid, nicotine, prurienidine, prurienine, riboflavin, saponins, serotonin, stizolamine, trypsin, tryptamine, vernolic acid, mucunadine, prurienine and prurieninine deliver synergistic effect to levodopa [31].

Preclinical studies suggest increased brain mitochondrial complex-I activity and restoration of endogenous levodopa, serotonin, and noradrenaline in the substantia nigra [32]. The neurorestorative effects of *M. pruriens *on the degenerating dopaminergic neurons in the substantia nigra are hypothesized to be due to increased complex-I activity, the presence of Nicotine adenine dinucleotide (NADH), and coenzyme Q-10. Genistein, gallic acid, unsaturated fatty acids, beta-carboline, phytic acid, and nicotine in *M. pruriens* possess neuroprotective activity [33-36]. Genistein also prevents peripheral decarboxylation of levodopa [37]. Studies also support the antioxidant activity of *M. pruriens* as established by increased levels of superoxide dismutase, glutathione (GSH), catalase, decreased lipid peroxidation, and mitochondrial permeability. Phytic acid present in *M. pruriens* is a non-toxic iron chelator with potent antioxidant activity [38,39]. GSH present in *M. pruriens* prevents neuronal loss and protects against free radical damage. Thus, secondary phytochemicals in *M. pruriens* other than levodopa may provide entourage-like effects in the management of PD.

Panchakarma (Bio-cleansing) in PD

The management of *vāta vyādhi* involves the employment of bio-cleansing procedures such as *vamana* (medical emesis), sudation, intestinal purgation, therapeutic enema, and nasal installations. Scholarly insight, such as that offered by Gourie-Devi et al. (2004), underscores that the treatment of *kampavāta* extends beyond mere herbal drug administration, instead encompassing a comprehensive regimen [40]. This regimen incorporates oral administration of medicated fats, massage employing medicated oils, sudation, induced purgation, application of medicated enemas, and errhine therapy. The pioneering exploration into the effects of *panchakarma* therapies within the context of PD was conducted by Nagashayana et al., who found that patients subjected to panchakarma followed by palliative care, in contrast to those undergoing solely palliative care, showed significant improvements in the activities in their daily lives and the motor examination [20]. In the case of PD, three procedures deserve special mention: *abhyanga* (massage), medicated** **enema, and nasal instillation of oil/ghee and aqueous liquids of herbs.

Abhyanga (Therapeutic Massage) Treatment in PD

Therapeutic body massage plays a pivotal role in the comprehensive management of *vāta vyādhi* [13]. Massage therapy in PD produces a multifaceted array of benefits. The treatment induces profound relaxation and effectively reduces stress hormone levels in urine (epinephrine and norepinephrine), enhancing the overall quality of life. The synergy of massage therapy with conventional medication yields tangible improvements in gait speed and subjective PD symptoms as evaluated on the Visual Analog Scale. It can potentially elevate health-related quality of life significantly [41]. Massage modalities, such as classical deep therapeutic massage, Thai massage, and neuromuscular therapy, have demonstrated their efficacy in ameliorating both motor and non-motor symptoms, such as sleep disturbances, pain, fatigue, anxiety, and depressive symptoms associated with PD [41]. Within the realm of Ayurveda, specific therapeutic oils prescribed for massage in vāta vyādhi, particularly in the context of neurological conditions like PD, have shown promise. Notable examples include the medicated oils mahā māṣa taila, dasamūla balā taila, and māṣādi taila, which have played pivotal roles in diverse case studies aimed at managing PD [42].

Basti (Medicated Enema) Treatment in PD

Emena is the primary therapy for effectively managing ailments brought about by the imbalanced *vāta doṣa*. This involves rectal administration of a decoction of herbs in an emulsion form, alternating with medicated oil enema. Medicated enema exerts broad therapeutic action on almost all body tissues and possesses rejuvenating, curative, preventive, and health-promotive measures [43]. Studies illustrate growing evidence of an altered microbiome in several neurodegenerative disorders, in particular, PD. Keshavarzian et al. reported a significant presence of "pro-inflammatory" protobacteria of the genus *Ralstonia *and a reduction in "anti-inflammatory" butyrate-producing bacteria from the genera *Blautia*, *Coprococcus*, and *Roseburia* in PD [44]. Studies further provide evidence of "pro-inflammatory" dysbiosis, which could trigger inflammation-induced misfolding of α-Syn and the development of PD [45]. Hegelmaier et al. reported that dietary intervention and bowel cleansing may provide a non-pharmacologic therapeutic option for PD patients [46]. In their case-control study assessing gut microbiome, 10 patients who received oil enema (water with electrolytes and 120 ml oil) and dietary intervention showed significant improvement in UPDRS III and decreased levodopa daily dose. Additionally, they observed a significant association between the gut microbiome diversity and the UPDRS III, the abundance of *Ruminococcaceae*, and a substantial reduction in *Clostridiaceae *after enema [46]. Āyurveda classics recommend enema in varied doses, patterns, and duration [43]. Several randomized uncontrolled open-label clinical trials and case studies have employed *mātra basti* (oil enema with the dose of 2 pala ~ 98 ml) wherein medicated oils *mahāmāṣa taila, māṣadi taila, and bala taila* have been used for the management of PD [47]. 

Nasya (Errhine) Treatment in PD

*Nasya* is a treatment procedure involving medicated oil or aqueous solution medicine administered through the nostrils. Ayurveda treatises consider the nose as the gateway to the head and indicate that medications installed through the nose reach the target site in the head and help prevent and manage various disorders of the head and neck [48]. Generally, it is known that nasal drug delivery directly targets the CNS, thus minimizing the systemic side effects. It aids in the delivery of drugs to the CNS, bypassing the blood-brain barrier (BBB). BBB allows lipid-soluble molecules to transport across the membrane. Therapeutic agents, including molecules and macromolecules, can be delivered to the CNS through the intranasal route. Olfactory and trigeminal neuronal connections between the nasal mucosa and brain provide a unique non-invasive pathway for delivering therapeutic agents to the CNS. Several enzymes, such as cytochrome P-450 enzyme isoforms, carboxylesterases, and glutathione S-transferases present in the nasal cavity, may play a role in the metabolism of drugs. The olfactory epithelium is a significant pathway for substances entering the CNS, and the extra-neuronal olfactory way capacitates the delivery of drugs directly to the brain parenchymal tissues and CSF through perineural channels. Although evidence of intra-nasal drug delivery in PD is limited, case studies involving Ayurveda management of PD have employed *nasya* with kṣīra balā taila, aṇu taila, māṣadi kvātha (decoction) along with oral *M. pruriens* and have observed substantial improvements in PD symptomatology [49].

Limitations of Studies in the Field

Though *M. pruriens* has shown anti‐Parkinsonian activity with less dyskinesia potency in open-label clinical and uncontrolled clinical trials involving heterogeneous PD patients [18,20,21], these studies lacked validated outcomes. They lacked vital methodological steps like randomization and blinding. Moreover, uncontrolled trials cannot exclude placebo effects and offer potentially biased results. The double-masked, crossover randomized trials were able to show significant improvements in *M. pruriens* over levodopa pharmacokinetics with validated outcome measures in terms of faster ON effects and lesser dyskinesia [22-24]. However, it should be emphasized that these studies included small samples of patients who were exposed to an acute dose of the tested medication, with no long-term follow-up and assessment of their response to *M. pruriens*. Studies involving Ayurveda *panchakarma* therapies were non-randomized, uncontrolled, lacked validated outcomes, failed to give a rationale behind the selection of the drugs, and could not explain the mode of action of the selected intervention [19,20]. 

Furthermore, the studies did not incorporate any neuroimmune, neuroimaging, or neurophysiological parameters to evaluate the neurobiological underpinnings of these interventions. Recommendations emphasize that clinical trials to assess therapeutic efficacy should adopt a double-masked, parallel, controlled design comprising a homogenous study group and utilize standardized measures and validated outcomes [50]. So far, no such trials are available for Ayurveda drugs and* panchakarma* therapies on PD. Although potential evidence on the efficacy of *M. pruriens* in treating PD is available, evidence can be considered insufficient for establishing the compound's effectiveness in preventing the progression and long-term management of PD.

## Conclusions

The neuroprotective and cognition-enhancing potential of Ayurveda offers much scope for neurodegenerative disorders like PD, offering significant benefits in motor and non-motor symptoms. Studies with *M. pruriens* show that it exhibits acute efficacy in patients with PD, but long-term follow-up and assessments are unavailable; the evidence is insufficient to reach any reliable conclusion. Further, identifying the *dosa* predominance, Hoehn & Yahr staging, and formulating appropriate measures depending on the staging is essential to individualize the treatment.

Ayurvedic interventions such as *M. pruriens *and *panchakarma* therapies might be effective in the management of PD. However, there is a need for methodologically rigorous randomized controlled clinical studies with adequate sample sizes. Such studies should incorporate standardized treatment schedules, long-term follow-up, and objective measures like multimodal neuroimaging and neurophysiological techniques to show both efficacy and possible mechanisms of action. 
